# Analysis of government investment in primary healthcare institutions to promote equity during the three-year health reform program in China

**DOI:** 10.1186/1472-6963-13-114

**Published:** 2013-03-25

**Authors:** Xiaopeng Zhang, Yuqi Xiong, Jing Ye, Zhaohua Deng, Xinping Zhang

**Affiliations:** 1 School of Medicine and Health Management, Tongji Medical College, HuaZhong University of Science and Technology, Wuhan, HuBei Province, China

## Abstract

**Background:**

The World Health Report 2000 stated that increased public financing for healthcare was an integral part of the efforts to achieve equity of access. In 2009, the Chinese government launched a three-year health reform program to achieve equity of access. Through this reform program, the government intended to increase its investment in primary healthcare institutions (PHIs). However, reports about the outcome and the improvement of the equity of access have yet to be presented.

**Methods:**

Stratified sampling was employed in this research. The samples used for the study comprised 34 community health service centers (CHSCs) and 92 township hospitals (THs) from six provinces of China. Collected data, which were publicly available, consisted of the total revenue, financial revenue, and the number of people for the periods covering January 2010 to September 2010 and January 2011 to September 2011. Revenue information for 2009 and 2010 was obtained from China’s Health Statistics Yearbook.

By using indicators such as government investment, government finance proportion and per capita revenue, t-tests for paired and independent samples were used to analyze the changes in government investment.

**Results:**

Government invest large amount of money to the primary healthcare institutions. Government finance proportion in 2008 was 18.2%. This percentage increased to 38.84% in 2011, indicating statistical significance (p = 0.000) between 2010 and 2011. The per capita financial input was 20.92 yuan in 2010 and 31.10 yuan in 2011. Compared with the figures from 2008 to 2010, the gap in different health sectors narrowed in 2011, and differences emerged. The government finance proportion in CHSCs revenue was 6.9% higher than that of THs, while the per capita revenue of CHSCs was higher. In 2011, the highest and lowest government finance proportions were 48.80% (Shaanxi) and 19.36% (Shandong), respectively. In that same year, the per capita revenue of Shaanxi (40.69 Yuan) was higher than that of Liaoning (28.79 Yuan). Comparing the 2011 figures with those from 2008 to 2010, the gap in 2011 clearly narrowed.

**Conclusion:**

In the three-year health reform program, the Chinese government increased its investment to PHIs gradually and significantly. Thus promote equity to access and universal coverage. However, the increase in government investment stemmed from political desire and from the lack of institutionalization of practice and experience. Hence, a mode of financial allocation must be formulated to promote consistency in government input after the three-year health reform program.

## Background

Health is a fundamental human right, declared by the WHO constitution of 1948, and promoting and protecting health is necessary to human welfare and sustained. WHO committed in 2005 and develop a health financing systems to ensure all people have access to health services without financial hardship, which was defined as universal coverage. Universal coverage means that everyone can use promotive, preventive, curative and rehabilitative health services when they need, of good quality and have no financial hardship.

In 2005, the 58^th^ World Health Assembly recommended that member states guarantee access to universal coverage, thereby achieving equity in access” [1].

Although policy makers have implemented several measures to achieve universal coverage, there still have barriers. The 2010 World Health statistics indicated that in 2007, general government expenditure on health accounts for 60.3% of the national health account on average, whereas the private expenditure contribution averaged 39.7% [2]. In China, the rate of the general government expenditure on health was 44.7%, which was lower than the private expenditure (55.3%). With the poor in the urban and rural areas of China at risk of being unable to afford healthcare services, there is a long way to universal coverage and equity to access.

So what can we do to achieve it? In fact, the World Health Report 2000 stated that increasing public financing for healthcare was an integral part of the efforts toward achieving universal coverage and equity [2]. More government input to healthcare, more skilled doctors can be treated, who can support better health service to patience. If the Government invests more to healthcare, especially to poor regions, the ratio of government financing in health expenditure will increase and the ratio of out-of-pockets will decrease. Thus, with increasing government input, people can have good quality and affordable health service, which promoting the equity of universal coverage.

Therefore, to promote fairness, the Chinese government launched a three-year implementation plan in 2009. The opinions of the CPC Central Committee and the State Council on widening the medical and health system reform indicated that the government input in health services would be gradually increased, while aiming to alleviate the burden of expensive medical bills of the citizens and increase their access to healthcare services [3,4]. The government promised to inject CNY 850 billion (US$ 124 billion) into the health care system in three years (2009–2011). Moreover, the government stated that increasing the input in primary healthcare institutions (PHIs) was one of the five top priorities this plan had explicitly set. In fact, in these three years, the government health investment amounted to about CNY 1409.9 billion (US$ 206 billion), and 44% of the funds were allocated for primary healthcare institutions according to a report from the Ministry of Finance in 2012 [5].

Primary healthcare institutions, including CHSCs and THs, are the essential parts of the healthcare system in China. The three-year plan pointed out that to ensure that patience to the primary healthcare institutions has good quality and affordable health service, at the same time, the health sectors can run smoothly, the government shall be responsible for its local primary healthcare institutions by taking on its national standard expenditure of basic development, procurement of equipment, and payment for medical faculty and public health care. In these three years, the primary healthcare institutions implemented the essential medicine policy. The fundamental drug zero rate differentia decreased the drug income by 36.5% to 62.9% [6]. Without the profit from drugs, government financial support seemed more significant to those institutions. However, how to compensate for the primary healthcare institutions remains unsolved [7].

In our study, we describe government investment and the promoting to equity of universal coverage. Then we analyze the differences of government investment between different provinces and between urban and rural areas. We attempt to give evidence of promoting to equity of government financing and identify the existing key problems. In the last, we give some suggestions to use government financing to promoting to equity.

## Methods

In our study, we assume that the deepening health reform, particularly in increasing government investment in primary healthcare institutions, improves the equity to access. Health sectors support health service and creating costs. The total revenue of health sectors is the money invested to them from government financing, health insurance and patience. And the total revenue of health sectors is the health expenditure taking place when there are activities of health service. In recent years, more and more people in China have health insurance and the ratio of health insurance in health expenditure increases. If the government (include central and local government) increase investment to health sector, it indicates efforts to achieve universal coverage. If the ratio of government in health expenditure increases, the ratio of out-of-pockets will decrease, thus promoting equity. So in our research, if the ratio of government financing in the total revenue increases, we say government increases the investment.

We employed stratified sampling for this study. We chose six provinces based on location and on the level of economic development. These provinces were Shandong and Liaoning province in the eastern region, Hubei and Shanxi province in the central region, as well as Shaanxi and Sichuan Province in the western region. In each province, we randomly selected 5 to 7 CHSCs and 15 to 17 THs, amounting to 34 CHSCs and 92 THs overall. We collected publicly available data consisting of the total revenue, financial revenue, and the number of people that the institutions covered from January 2010 to September 2010 and January 2011 to September 2011. We obtained the revenue information of the primary healthcare institutions from the 2009 and 2010 China Health Statistics Yearbook.

We use financial revenue as government’ investment, analyze government input to the primary healthcare institutions and the change from 2010 to 2011. Also indicators A and B (see definition below) are used to reflect the level of government’s increasing input.

### Definitions

#### Indicator A

$$\begin{array}{l} \;\text{Government}\;\text{finance}\;\mathrm{proportion}\\ =\frac{\text{Financial}\;\text{revenue}\;\mathrm{from}\;\mathrm{the}\;\mathrm{government}}{\text{Total}\;\text{revenue}\;\text{of}\;\text{the}\;\mathrm{primary}\;\mathrm{healthcare}\;\mathrm{institutions}} \end{array}$$

Indicator A shows the level of inputs of the government financing.

#### Indicator B

$$\begin{array}{l} \mathrm{Per}\;\mathrm{capita}\;\mathrm{revenue}\\ =\frac{\text{Financial}\;\text{revenue}\;\text{from}\;\mathrm{the}\;\mathrm{government}}{\mathrm{Population}\;\mathrm{of}\;\mathrm{the}\;\mathrm{covered}\;\mathrm{facilities}} \end{array}$$

As the populations the primary healthcare institutions covered were different, Indicator B indicates the average inputs on each person of government, in comparison with indicator A.

In obtaining indicators A and B, we used SPSS13.0 to analyze the increases and the differences in government investment to primary healthcare institutions from 2009 to 2011, the inequity of the six provinces, and the unfairness in the urban and rural areas. Paired sample *t* test and independent sample *t* test were used in our study.

## Results

### Changes of government financing inputs

From Table 1, we can see that the government (include central and local government) invests 881 thousand yuan on average to a primary healthcare institution in 2010. In 2011, the investment increases to 1300 thousand yuan. What’s more, this increasing occurs in all the six provinces we select. These indicate that the government makes effort to increasing government financing.

**Table 1:** Government financing input to primary healthcare institutions in 2010 and 2011 (thousand yuan)

**Province**	**2011**			**2010**		
	**Mean**	**Minimum**	**Maximum**	**Median**	**Minimum**	**Maximum**
Hubei	1747	130	7830	1244	30	5160
Liaoning	1139	50	6400	613	0	4139
Shandong	1806	410	5290	852	0	3380
Shanxi	549	30	2740	409	20	1920
Shaanxi	1225	90	2750	1102	100	2372
Sichuan	1239	0	13080	1034	37	12715
Average	1300	0	13080	881	0	12715

Table 2 shows that government finance proportion was 18.2% in 2008. This value has increased since the implementation of the three-year plan in 2009. In 2011, the proportion increased to 38.84%. From 2010 to 2011, the ratio increased by 8.98%, which was statistically significant (p = 0.000). From 2010 to 2011, the government finance proportion in Hubei, Liaoning, and Shandong increased by 9.96%, 13.3%, and 16%, respectively; the improvements were statistically significant (p values were 0.024, 0.001, and 0.000, respectively), as shown in Table 2.

**Table 2:** Government finance proportion in primary healthcare institutions, 2008–2011 (%)

**Province**	**A2008**	**A2009**	**A2010**	**A2011**	**A2011 − A2010**	**P values**
Hubei	—	—	33.27	43.23	9.96	0.024
Liaoning	—	—	27.19	40.32	13.13	0.001
Shandong	—	—	19.36	35.36	16.00	0.000
Shanxi	—	—	34.00	35.23	1.23	0.269
Shaanxi	—	—	48.80	53.40	4.60	0.218
Sichuan	—	—	29.50	32.15	2.65	0.262
Average	18.2	19.3	29.86	38.84	8.98	0.000
P values	—	—	0.000	0.074	—	—

Note: A stands for Indicator A.

From Table 3, we can see the government finance proportion has increased since 2008 in both CHSCs and THs. The CHSC ratio was 46.8%, and the TH ratio was 37.9%. These ratios indicate a rapid increase in government financial investments. In per capita revenue (Table 4), it improves in 2011 compared with 2010.

**Table 3:** Government finance proportion between urban and rural areas (%)

**Financing year**	**CHSCs (D)**	**THs(E)**	**D-E**	**P values**
A2011	46.8	37.9	6.9	0.978
A2010	34.2	25.1	9.1	0.451
A2009	21.1	18.8	2.3	—
A2008	20.7	17.5	3.2	—

Note: A stands for Indicator A.

**Table 4:** Statistics of per capita revenue among different provinces, January 2010 to September 2010, January 2011 to September 2011 (yuan/person)

**Province**	**B2011**	**B2010**	**B2011 − B2011**	**P values**
Hubei	34.93	24.88	10.05	0.076
Liaoning	28.79	15.49	13.30	0.000
Shandong	28.58	13.49	15.09	0.000
Shanxi	20.78	14.71	6.07	0.004
Shaanxi	40.69	36.62	4.07	0.079
Sichuan	32.91	27.48	5.43	0.007
Average	31.10	20.92	10.18	0.000
P	0.027	0.000	—	—

Note: B stands for Indicator B.

These figures reflect the increasing government financial investments in primary healthcare institutions increases the ratio increase the ratio of government financing in health expenditure.

### Differences of the government inputs between different provinces

The investment to the primary healthcare institutions is influenced by many factors, such economic conditions, need of health service and so on. We can not analyze equity of different provinces by the amount of money that government invests to the primary healthcare institutions. But the ratio of the government input in health expenditure can reflect the ratio of out-of-pockets, so we can use this to evaluate the promoting to equity of universal coverage.

Table 2 shows that Shaanxi (48.80%) received the highest government finance proportion, while Shandong had the lowest (19.36%) in 2010. A difference existed in the values among the provinces in 2010 (p = 0.000), but the difference disappeared in 2011 (p = 0.074). The results illustrate the improvement of equity of government financing among the different provinces in 2011 after the new medical reform.

However, from the absolute value in 2011, the highest rate was 53.40% in Shaanxi, while the lowest rate was 32.15% in Sichuan. These values indicate certain differences among the regions.

### Government inputs between urban and rural health sectors

As Table 3 shows, the government finance proportion in urban areas in 2010 and 2011 was greater than that in rural areas. In 2011, government finance proportion for CHSCs was 6.9% higher than that for THs, but the difference was not statistically significant (p = 0.978 in 2011, p = 0.451 in 2010). This finding indicates that the government financial investment between the urban and rural areas seemed relatively equal.

However, based on the absolute value, the gap between the urban and rural areas in 2009 was 2.3%, which was a decrease when compared with that in 2008. In 2010 and 2011, the gaps were 9.1% and 6.9%, respectively. The obvious increase in these figures when compared with those from 2008 indicates the widening gap in financial input between urban and rural areas.

As seen in Table 5, the per capita revenue in the urban areas was greater than that in the rural areas from January 2010 to September 2010 and from January 2011 to September 2011. However, the difference was not statistically significant (p values were 0.702 and 0.291, respectively). In 2011, per capita financial input in CHSC was 1.27 times greater than that in rural hospitals, which was 1.29 times lesser than that in the same period of 2010. This finding indicates the narrowed gap between urban and rural areas.

**Table 5:** Statistics of per capita revenue in different primary healthcare institutions, January 2010 to September 2010, January 2011 to September 2011 (yuan/person)

**Financing year**	**CHSCs (D)**	**THs (E)**	**D/E**	**P values**
B2011	35.7	28.1	1.27	0.291
B2010	24.2	18.8	1.29	0.702
B2011 − B2010	11.5	9.3	—	—
P values	0.002	0.000	—	—

Note: B stands for Indicator B.

## Discussion

### The promoting to equity of government financing in primary healthcare institutions

In the three-year reform program, the Chinese government increased input and reduced the medical revenue of primary healthcare institutions in some aspects, thereby reducing the proportion of out-pocket medical expenses to promote access and equity. In our research, we observed that the government invest lots of mony to the primary healthcare institutions and the government finance proportion reached 19.3% in 2009, which was sharply higher than the 8.3% in Uganda [8]. As is discussed above, the increase of the ratio of government financing in health expenditure can reflect the decrease of the ratio of out-of-pockets. Thus promotes the equity of universal coverage.

In China, the government financial compensation comes from the central government and the local government. However, a study showed that the budget of the central government for primary health care was zero, as it is mainly allocated for hospitals and other expenditures [9]. As a result, the increased government investment as stated in the reform program has become a burden for the local governments in China. In some poor areas, primary healthcare institutions do not work well if the inputs are too expensive for the local government. In turn, local people’s access to healthcare becomes ambiguous.

Unfortunately, simply raising more government funds is not enough to achieve corresponding goals if the healthcare system is too weak to support the rapid scale-up of service coverage [10,11]. In China, the government restricts the budget to the health sectors. With this restriction, along with the lack of effective implementation of good performance practices, some primary health sectors in some provinces are wasteful and inefficient

Therefore, in the case of the coexistence of the limited capacity of local financial input and waste, the government is faced with the problem of ensuring that limited health expenditure plays a role in reducing the burden of health expenditure for citizens, and of improving access to equity.

### The equity between different provinces and regions

The proportion of government spending allocated to the health sector provides important insights into the value that governments place on health [12]. In China, from the view of governmental financing to primary healthcare institutions, more governmental investments promoted equity in different regions, narrowing the gap between urban and rural areas. However, differences still exist between urban and rural areas in per capita government compensation. The inequity may be due to the geographical location, as suggested by several researchers [13].

In China, the service capabilities of primary healthcare institutions and the population they cover have yet to reach a relatively appropriate proportion, and the government financial compensation remains unclear. Thus, promoting the equity of the usage in healthcare resource between different locations is difficult.

The embarrassing situation of healthcare equity in China is similar to what once happened in Britain [14]. To achieve equity in health expenditure, the British government announced that financial allocation to an area should be based on five variables. These variables were as follows: standardized limiting long-standing illness ratio (age less than 75 years), standardized mortality ratio (age less than 75 years), proportion of economically active citizens who are unemployed, proportion of pensionable-aged citizens living alone, and proportion of dependents in single-carer households. In contrast, China has no clear basis.

Other studies emphasized that governments should ensure that resources are allocated according to need [4]. The WHO indicates that funding needs vary with differences in costs, population age structures, and patterns of diseases. Dealing with universal health coverage means dealing with the poor and the marginalized, as well as with people who are often politically disenfranchised and lack representation [12]. Developing equitable financing approaches depend on the assessment of the burden and the determinants of out-of-pocket funding on healthcare sought by different socio-economic and geographic groups, which leads to determining how best to protect the poor. In addition, equity can be achieved only through public expenditures targeted to the poorer and underserved sections of the society [15]. Therefore, creating a set of modes that determine the government financial expenditure for health care facilities by including relative factors is necessary. Factors that should be considered include economic performance, average income of residents, service population of health facilities, etc. Based on the experience of the WHO and developed countries, the Chinese government should allocate pooled resources among geographical regions that account for relative population sizes, income/poverty levels, health needs, and unavoidable differences in the costs of delivering services (e.g., due to low population density) [16]. Such action will improve the inequity in different health sectors of different provinces.

### Consistency of government input

The strengthening of governmental expenditure during the past three years has promoted the equity of healthcare. However, this development is apparently a result of a political mandate. As 2011 was the last year of the three-year plan, the consistency of the policy after 2011 is still unknown, considering the ability of the government to bear more healthcare expenditure. Various authors highlight the need to harmonize healthcare financing arrangements. They note that health financing should be approached from a systemic perspective that is informed by policy goals instead of implementing piece-meal reforms that focus on each source of funding independently, which in turn promotes fragmentation and segmentation [17-19]. Clearly, the past and current health reforms in China have not adopted a systemic approach to healthcare financing. Without substantial progress, prospects of promoting accountability and equity of the health system are slim [20]. Thus, the consistency in medical reform in China must be guaranteed.

## Conclusion

New medical reforms stimulated government financial input in primary healthcare institutions. The influx of financial input improved the current situation of the expenditure fairness in different provinces and regions. However, the increase in government inputs is the result of political desire during the three-year health reform program. The lack of the institutionalization of practice and experience is apparent. Moreover, the system and standards of government financial input are unclear, and the inequity of government financing allocation and pursing still exists. Therefore, the establishment of a government financing allocation mechanism is extremely important. In addition, after the three-year new medical reform, China faces the problem of ensuring the sustainability of the system of the health reform program to achieve equity to access and universal coverage.

## Abbreviations

WHO: World Health Organization; PHIs: Primary Healthcare Institutions; CHSCs: Community Health Service Centers; THs: Township Hospitals.

## Competing interests

The authors declare no competing interests.

## Authors’ contributions

Xinping Z designed the study. Xiaopeng Z conducted the health economics studies, performed the statistical analysis, and drafted the manuscript. JY, YX, and ZD participated in the writing of the manuscript. All authors read and approved the final manuscript.

## Pre-publication history

The pre-publication history for this paper can be accessed here:

http://www.biomedcentral.com/1472-6963/13/114/prepub
